# Betaine Treatment Attenuates Chronic Ethanol-Induced Hepatic Steatosis and Alterations to the Mitochondrial Respiratory Chain Proteome

**DOI:** 10.1155/2012/962183

**Published:** 2011-12-08

**Authors:** Kusum K. Kharbanda, Sandra L. Todero, Adrienne L. King, Natalia A. Osna, Benita L. McVicker, Dean J. Tuma, James L. Wisecarver, Shannon M. Bailey

**Affiliations:** ^1^Research Service-151, Veterans Affairs Nebraska-Western Iowa Health Care System, 4101 Woolworth Avenue, Omaha, NE 68105, USA; ^2^Department of Internal Medicine, University of Nebraska Medical Center, Omaha, NE 68198, USA; ^3^Department of Environmental Health Sciences, University of Alabama at Birmingham, Birmingham, AL 35294, USA; ^4^Department of Pathology and Microbiology, University of Nebraska Medical Center, Omaha, NE 68198, USA

## Abstract

*Introduction*. Mitochondrial damage and disruption in oxidative phosphorylation contributes to the pathogenesis of alcoholic liver injury. Herein, we tested the hypothesis that the hepatoprotective actions of betaine against alcoholic liver injury occur at the level of the mitochondrial proteome. *Methods*. Male Wister rats were pair-fed control or ethanol-containing liquid diets supplemented with or without betaine (10 mg/mL) for 4-5 wks. Liver was examined for triglyceride accumulation, levels of methionine cycle metabolites, and alterations in mitochondrial proteins. *Results*. Chronic ethanol ingestion resulted in triglyceride accumulation which was attenuated in the ethanol plus betaine group. Blue native gel electrophoresis (BN-PAGE) revealed significant decreases in the content of the intact oxidative phosphorylation complexes in mitochondria from ethanol-fed animals. The alcohol-dependent loss in many of the low molecular weight oxidative phosphorylation proteins was prevented by betaine supplementation. This protection by betaine was associated with normalization of SAM : S-adenosylhomocysteine (SAH) ratios and the attenuation of the ethanol-induced increase in inducible nitric oxide synthase and nitric oxide generation in the liver. *Discussion/Conclusion*. In summary, betaine attenuates alcoholic steatosis and alterations to the oxidative phosphorylation system. Therefore, preservation of mitochondrial function may be another key molecular mechanism responsible for betaine hepatoprotection.

## 1. Introduction

Chronic ethanol exposure has been shown to significantly alter liver mitochondrial structural and functional integrity. Ethanol consumption alters mitochondrial morphology, induces mitochondrial DNA damage, and impairs ribosomal activity and structure [1–4] resulting in depressed mitochondrial protein synthesis and associated loss of electron transport chain complexes levels and function. It has also been shown that alcohol exposure increases the sensitivity of liver mitochondria to induce mitochondrial permeability transition pore [5] that may be linked to higher cyclophilin D levels in liver mitochondria [6]. Together, these chronic ethanol-induced alterations result in depressed respiratory capacity and impaired oxidative phosphorylation, events critical to the development of alcoholic liver injury [7–10].

In recent years, advancements in proteomic technologies have facilitated the examination of alcohol-dependent alterations to the mitochondrial proteome [11]. Using both conventional and blue native (BN)-PAGE proteomics methods, Bailey et al. have reported that ethanol exposure results in the decrease of both nuclear and mitochondrial encoded gene products of the oxidative phosphorylation system [11]. Similar defects in the mitochondrial proteome such as reductions in cytochrome *c* oxidase subunits and mitochondrial membrane potential, have also been reported in genetically altered mice exhibiting deficiency in liver levels of SAM [12], buttressing the concept that SAM plays a critical role in maintaining proper mitochondrial function.

Several studies including ours have demonstrated that while the alcohol-induced decrease in hepatic SAM levels is detrimental, it is the decreased hepatocellular SAM : S-adenosylhomocysteine (SAH) ratio that adversely affects many crucial SAM-dependent methylation reactions and the ultimate generation of many hallmark features of alcoholic liver disease [13–16]. We have further shown that the addition of SAM can normalize alcohol-induced SAM : SAH ratios [17] and preserve mitochondrial respiratory capacity by maintaining the mitochondrial genome and proteome while attenuating alcohol-dependent increases in mitochondrial superoxide production [3, 18].

Betaine, a methyl donor and another key metabolite of the methionine cycle, has been shown to normalize hepatocellular SAM : SAH ratio, correct defective cellular methylation reactions, and prevent the alcohol-mediated steatosis, apoptosis, and accumulation of damaged proteins [14, 17, 19–23]. Based on this, we investigated whether betaine prevents alcohol-induced changes to the mitochondrial oxidative phosphorylation system in a rat model of chronic alcohol exposure. This assessment was complemented by determinations of liver cytochrome P450 2E1 (CYP2E1) protein and activity, glutathione (GSH) levels, SAM : SAH ratios, NOS2 expression, NO generation, and triglyceride levels.

## 2. Materials and Methods

### 2.1. Diet Formulation

Nutritionally adequate Lieber-DeCarli control and ethanol liquid diets [24] were purchased from Dyets, Inc. (Bethlehem, Pa, USA). The ethanol diet consisted of 18% of total energy as protein, 35% as fat, 11% as carbohydrate, and 36% as ethanol. In the control diet, ethanol was replaced isocalorically with carbohydrate such that both ethanol and control rats consumed identical amounts of all nutrients except carbohydrate.

### 2.2. Ethanol and Betaine Feeding Procedure

Male Wistar rats (Charles River Laboratories, Wilmington, Mass, USA) weighing 180 to 200 g (approximately 45–48 days old) were weight-matched and divided into four groups. Group 1 was fed the control diet. Group 2 was fed the same diet as Group 1 except 1% (w/v) betaine was added to the diet. Group 3 was fed the ethanol diet, and Group 4 was fed the ethanol diet containing 1% (w/v) betaine. Rats in groups 1–3 were fed the amount of diet consumed by rats in group 4. Overall, each group consisted of 8 rats fed the appropriate diet for 4-5 weeks. Twenty four hours before sacrifice, the total daily volume of the diet was divided with 1/4 given at 8:00 am, 1/4 at 12:00 noon, and 1/2 at 4:00 pm. In addition, animals were given 1/4 their respective diets 60–90 minutes prior to death. This regimen was followed to minimize differences in feeding patterns that exists between the groups of rats. The care, use, and procedures performed on these rats were approved by the Institutional Animal Care and Use Committee at the Omaha Veterans Affairs Medical Center and complied with NIH guidelines.

### 2.3. Liver Histology and Detection of Lipid Accumulation

Formalin fixed liver tissue was processed for hematoxylin-eosin staining and evaluated for steatosis and inflammation. In addition, fresh frozen liver sections were fixed in 4% w/v paraformaldehyde in 50 mM PIPES, pH 7.0, and the accumulated lipid were visualized by staining with 1 *μ*g/mL BODIPY 493/503 (Invitrogen, Carlsbad, Calif, USA). After incubation, slides were washed twice with PBS and mounted with “Vectashield with DAPI” (Vector laboratories, Burmingham, Calif, USA). Images were obtained with a Zeiss 510 Meta Confocal Laser Scanning Microscope using an excitation wavelength of 488 nm and an emission wavelength of 505 nm.

### 2.4. Triglycerides

Total lipids were extracted from the liver to quantify the triglyceride mass using the triglyceride diagnostics kit (Thermo DMA kit, Thermo Electron Clinical Chemistry, Louisville, Colo, USA) as detailed in our publication [14].

### 2.5. Mitochondria Isolation

Pieces of fresh liver were homogenized in cold 5 mmol/L Tris (pH 7.4) containing 0.25 mol/L sucrose and 1 mmol/L EDTA, and liver mitochondria were isolated by differential centrifugation techniques [3].

### 2.6. Blue Native Gel Electrophoresis

Ethanol and/or betaine effects on the levels of mitochondrial proteins that comprise the oxidative phosphorylation system were assessed using BN-PAGE proteomics as detailed [3]. Image analysis on two-dimensional BN-PAGE gels was performed using Quantity One software (Bio-Rad Laboratories, Hercules, Calif, USA).

### 2.7. SAM, SAH, and GSH Levels

The perchloric acid extract of total liver was filtered through a 0.22 *μ*m membrane filter and directly subjected to HPLC analysis for the determination of SAM, SAH, and GSH levels, as detailed [14]. GSH levels in the mitochondrial fractions were also determined.

### 2.8. CYP2E1 Activity

The activity was measured in liver homogenates by the formation of 4-nitrocatechol (4-NC), as previously described [25]. CYP2E1 specific activity is expressed as nmol 4-NC produced per hr per mg protein.

### 2.9. NOS2 Gene Expression

NOS2 gene expression was determined by real-time quantitative PCR. Total hepatic RNA was extracted, treated with DNase I (Invitrogen, Carlsbad, Calif, USA) and used to synthesize cDNA using Taqman Reverse Transcription reaction kits (Applied Biosystems, Foster City, Calif, USA). After amplification, real-time quantitative PCR was performed using an Applied Biosystems 7500 Real-Time PCR System and Taqman Assay-on-Demand gene expression assays for rat NOS2 and *β*-actin (housekeeping gene) according to the manufacturer's instructions. The comparative Ct method was used to determine the relative concentration of the RNA transcript and the result expressed as “fold change” relative to the housekeeping gene.

### 2.10. Western Blotting

Immunoblots were performed by loading equal amounts of homogenate or mitochondrial proteins onto SDS-PAGE gels. Isolated liver mitochondria were used to detect mitochondria proteins, while total liver homogenates were used for examining inducible nitric oxide synthase (NOS2) and CYP2E1. Levels of NOS2 protein were detected using a 1 : 1,000 dilution of antibody (BD/Pharmingen, San Diego, Calif, USA). CYP2E1 protein was detected using a 1 : 2,000 dilution (Calbiochem, Gibbstown, NJ, USA). Cytochrome *c *oxidase subunit I was detected using 1 : 5,000 dilution (Molecular Probes, Eugene, Ore, USA). After membranes had been incubated with the appropriate secondary antibodies, proteins were visualized using standard enhanced chemiluminescence detection methods. The intensities of immunoreactive protein bands were quantified using Quantity One software (Bio-Rad Laboratories, Hercules, Calif, USA).

### 2.11. Mitochondrial NO

Levels of nitrates and nitrites (the end product of nitric oxide, NO) were measured in the mitochondrial fraction using the Griess reaction as detailed [26].

### 2.12. Statistical Analysis

Data were analyzed by ANOVA, followed by Student's Newman-Keuls post hoc test. A *P* value <0.05 was regarded as statistically significant.

## 3. Results

### 3.1. Liver Histopathology, Triglycerides, and SAM : SAH Ratios

**Figure 1:**
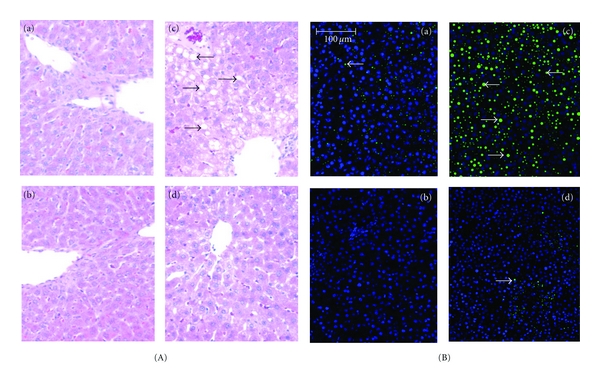
Betaine attenuates chronic ethanol-dependent steatosis. Hematoxylin-eosin (A) and BODIPY 493/503-DAPI (B, green BODIPY-labeled lipid droplets, nuclei blue) stained images from representative livers of (a) control; (b) control + betaine; (c) ethanol; (d) ethanol + betaine rats (*n* = 8 rats'/group) fed the respective diets for 4-5 weeks. Liver sections of the representative ethanol-fed rat stained with hematoxylin-eosin or BODIPY 493/503-DAPI shows micro- and macrovesicular steatosis (arrows).

**Figure 2:**
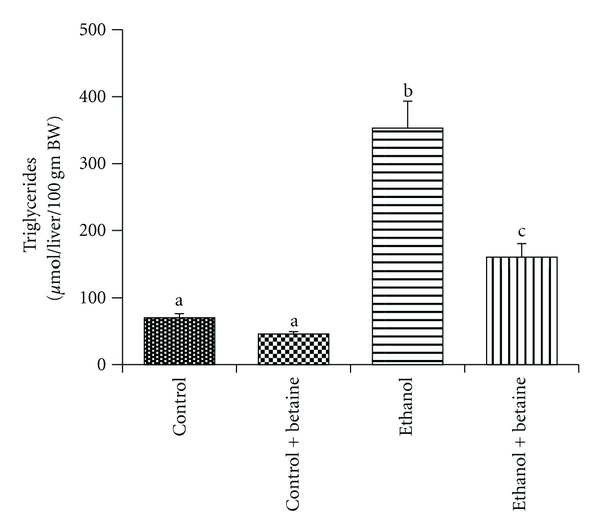
Betaine attenuates chronic ethanol-mediated increase in hepatic triglyceride levels. Triglyceride content in the liver lipid extract was quantified using the diagnostics kit (Thermo Electron Clinical Chemistry, Louisville, Colo, USA). Data represent the mean ± S.E.M. for *n* = 8 animals per treatment group. Values not sharing a common letter are statistically different, *P* < 0.05.

The histopathological evaluation of livers within each group were consistent with our previously published data [14]. Livers from the rats fed ethanol for 4-5 weeks displayed micro- and macrovesicular steatosis; however, no steatosis was observed in livers of rats fed the betaine-supplemented ethanol diet (Figure 1). Indeed, these livers showing similar histology as the livers of control or the betaine-supplemented control rats (Figure 1). Visualization of lipid droplets using green fluorescent BODIPY 493/503 showed considerable accumulation of neutral lipids, including esterified cholesterol in ethanol-fed rat livers (Figure 1). Minimal lipid accumulation was observed in the controls, or betaine-supplemented ethanol fed-rat livers (Figure 1). Biochemical analysis of the liver triglycerides levels corroborated the histopathology and neutral fat staining results. A significant attenuation of hepatic triglyceride content was observed in rats fed the betaine-supplemented ethanol diet as compared to the rats fed ethanol alone (Figure 2).

**Figure 3:**
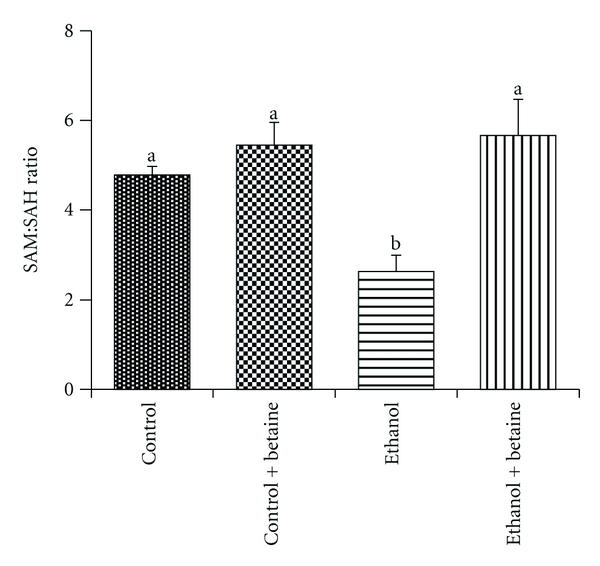
Betaine prevents the chronic ethanol-dependent decrease in the hepatic SAM : SAH ratio. Liver SAM and SAH levels were determined by HPLC analysis, and the SAM : SAH ratio was calculated as previously described [14]. Data represent the mean ± S.E.M. for *n* = 8 animals per group. Values not sharing a common letter are statistically different, *P* < 0.05.

Ethanol consumption for 4 weeks had no effect on hepatic SAM levels, but dramatically increased SAH levels [13, 14], resulting in a lower SAM : SAH ratio as compared with controls (Figure 3). Feeding rats a betaine-supplemented control or ethanol diet increased hepatic SAM levels 3- and 6-fold, respectively (data not shown). SAH levels followed the same pattern as SAM in these two groups (data not shown). These relative changes in the levels of SAM and SAH in both betaine-supplemented groups resulted in comparable hepatic SAM : SAH ratios as in the controls. These results were similar to our previous observations [14].

### 3.2. Liver NOS2 and NO Generation

**Figure 4:**
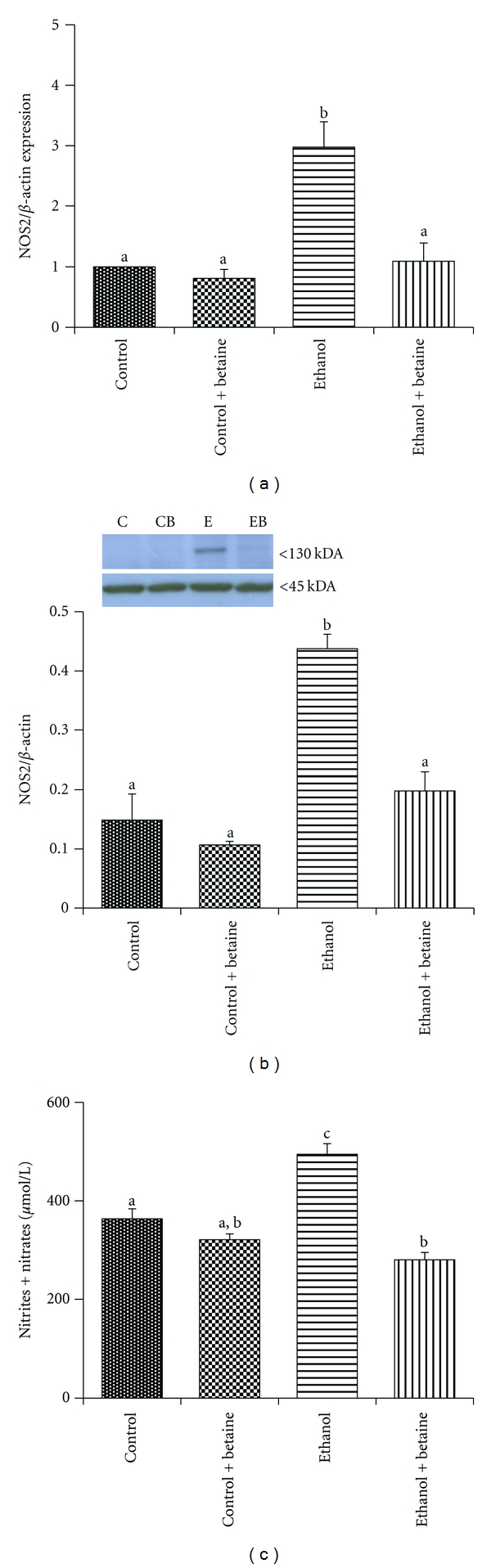
Betaine attenuates chronic ethanol-induced increase hepatic NOS2 gene and protein expression and elevates mitochondrial nitrite/nitrate levels. (a) Liver NOS2 mRNA levels were measured by quantitative PCR using the comparative Ct concentration method. The data shown are mean ± SEM of eight determinations from each group. Values not sharing a common subscript letter are statistically different, *P* < 0.05. (b) Hepatic NOS2 (130 kDa) protein expression was measured by immunoblotting and normalized to *β*-actin (45 kDa). Representative immunoblots of NOS2 protein for one pair of untreated and one pair of betaine-treated control and ethanol animals is shown. The bar graph results below represent the mean volume integration units (V.I.U) of NOS2 ± S.E.M. for *n* = 8 animals per group. Values not sharing a common letter are statistically different, *P* < 0.05. (c) Total mitochondrial nitrite and nitrate levels were measured by the Griess reaction as detailed [26]. The bar graph results represent the mean ± S.E.M. for *n* = 8 animals per group. Values not sharing a common letter are statistically different, *P* < 0.05.

Chronic ethanol consumption caused induction of NOS2 both at the gene expression and the protein level (Figures 4(a) and 4(b), resp.), which is in agreement with other reports [27]. As a consequence of NOS2 induction, increased levels of nitrite/nitrate, byproducts of nitric oxide metabolism, were detected in mitochondrial fractions of ethanol-fed rats (Figure 4(c)). Interestingly, the ethanol-dependent increase in hepatic NOS2 and nitrite/nitrate levels were attenuated by supplementation of the ethanol diet with betaine (Figures 4(a)–4(c)).

### 3.3. Liver CYP2E1 Activity and Protein Levels

**Figure 5:**
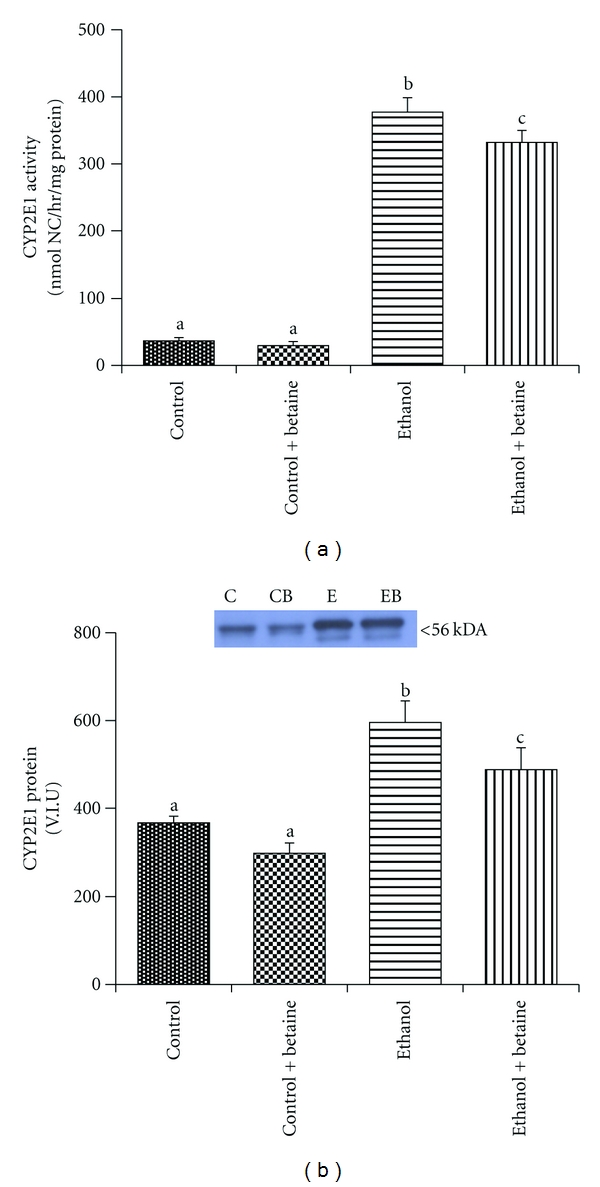
Betaine has minimal effect on chronic ethanol-dependent increase in CYP2E1 activity and protein. (a) Hepatic CYP2E1 activity was determined as detailed in Section 2. Data represent the mean ± S.E.M. for *n* = 8 animal per groups. Values not sharing a common letter are statistically different, *P* < 0.05. (b) CYP2E1 protein levels were measured by immunoblotting. The bar graph results represent the mean volume integration units (V.I.U) ± S.E.M. for *n* = 8 animals per group. Values not sharing a common letter are statistically different, *P* < 0.05. The top figure shows representative immunoblots of CYP2E1 protein for one pair of untreated and one pair of betaine-treated control and ethanol animals.

Chronic ethanol consumption induced a 10-fold increase in CYP2E1 activity (Figure 5(a)). This increase in activity was also reflected by an induction in CYP2E1 protein level (Figure 5(b)). A marginal, but statistically significant, decrease (~12%) in both CYP2E1 protein and activity was observed in livers of rats fed the betaine-supplemented ethanol diet (Figures 5(a) and 5(b)).

### 3.4. Mitochondrial GSH Levels

**Figure 6:**
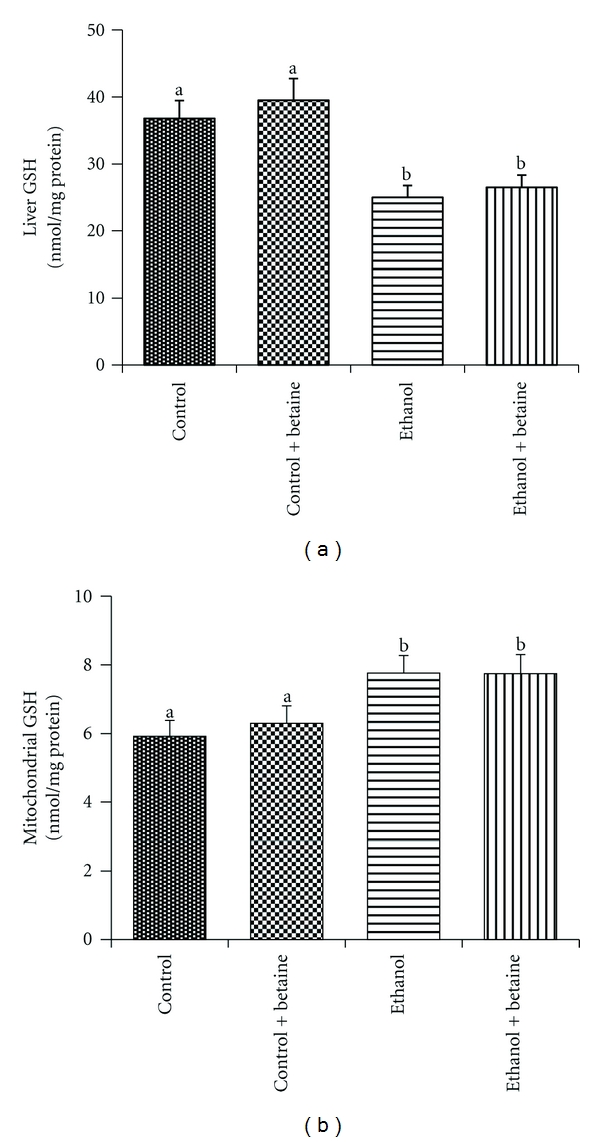
Betaine and ethanol increase mitochondrial GSH levels. HPLC was employed to determine GSH levels in (a) liver and the (b) mitochondrial fractions from control, ethanol, control + betaine, or ethanol + betaine groups. Data represent the mean ± S.E.M. for *n* = 8 animals per group. Values not sharing a common letter are statistically different, *P* < 0.05.

While chronic ethanol consumption decreased total liver GSH in the current study (Figure 6(a)), mitochondrial GSH was increased following chronic ethanol consumption (Figure 6(b)). Betaine treatment had no effect on the ethanol-induced decrease in total liver GSH or increased mitochondrial GSH levels observed (Figures 6(a) and 6(b)).

### 3.5. Assessment of Oxidative Phosphorylation Proteins

**Figure 7:**
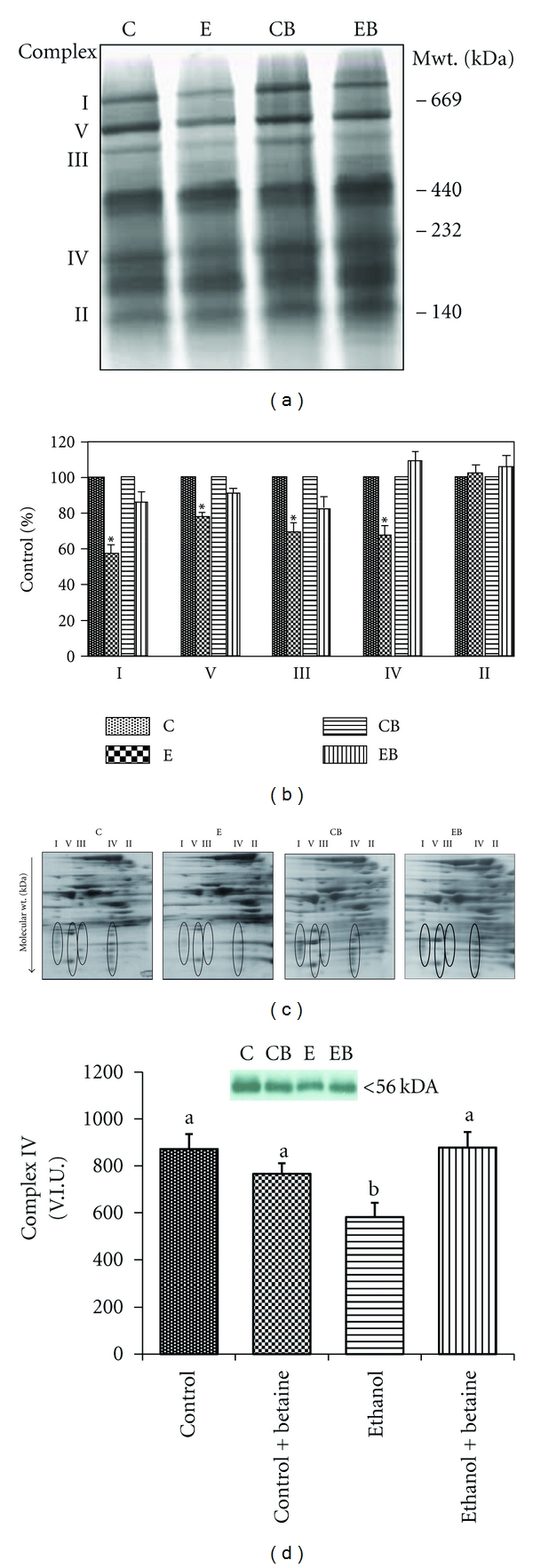
Betaine prevents the chronic ethanol-mediated loss in oxidative phosphorylation proteins. Representative (a) one- and (c) two-dimension blue native (BN)-PAGE proteomic gels of mitochondria isolated from rats fed control, ethanol, control + betaine, or ethanol + betaine diets as detailed in Section 2. (a) For these gels, 250 *μ*g of mitochondrial protein were subjected to 1D BN-PAGE. (c) The 1D gel strips were overlaid across a Tris-Tricine-SDS-PAGE gel to resolve the individual polypeptides that comprise each oxidative phosphorylation system complex. The proteins that comprise each complex (I, V, III, IV, and II) appear as vertically aligned spots on the 2D gel. (b) Comparison of the relative quantities of complexes I, V, III, and IV in liver mitochondria from the control; control + betaine; ethanol, and ethanol + betaine. Statistical analyses for data presented in panel B: 2-factor ANOVA on raw densitometry values—complex I: ethanol *P* = 0.0002, betaine *P* = 0.92, interaction *P* = 0.02; complex V: ethanol *P* = 0.016, betaine *P* = 0.72, interaction *P* = 0.23; complex III: ethanol *P* = 0.005, betaine *P* = 0.92, interaction *P* = 0.31; complex IV: ethanol *P* = 0.15, betaine *P* = 0.62, interaction *P* = 0.045; complex II: ethanol *P* = 0.54, betaine *P* = 0.98, interaction *P* = 0.80. (d) Complex IV, subunit 1 protein levels were measured by immunoblotting. The bar graph results represent the mean volume integration units (V.I.U) ± S.E.M. for *n* = 8 animals per group. Values not sharing a common letter are statistically different, *P* < 0.05. The top figure shows representative immunoblots of subunit 1 protein for control, ethanol, and betaine supplemented control and ethanol-fed rats.

Examination of oxidative phosphorylation proteins by BN-PAGE revealed that betaine prevented an ethanol-induced loss of oxidative phosphorylation proteins. Representative one- and two-dimension BN-PAGE proteomic maps are shown in Figures 7(a) and 7(c). Similar to our previously published data [3], the two-dimensional proteomic maps revealed a loss of respiratory chain proteins following chronic ethanol exposure. This was more apparent for proteins that comprise cytochrome *c* oxidase (i.e., complex IV) and the NADH dehydrogenase (i.e., complex I) (Figure 7(b)). Supplementation of the ethanol diet with betaine significantly attenuated the ethanol-induced loss in these mitochondrial proteins. The effects of ethanol and betaine were also reflected in Western blot analysis of complex IV. Representative data for complex IV is shown in Figure 7(d). For example, the ethanol-dependent loss in complex IV subunit I was attenuated by the inclusion of betaine in the ethanol diet.

## 4. Discussion

The role of mitochondrial dysfunction in the pathogenesis of alcoholic liver disease has long been documented by multiple laboratories [2–4, 7–10]. Mitochondria are a recognized source of reactive oxygen and reactive nitrogen species following ethanol consumption and are also a key target of subsequent oxidative posttranslational modifications including components of the oxidative phosphorylation system [10]. Recent studies using BN-PAGE proteomics, show that chronic ethanol consumption decreases the levels of several nuclear and mitochondrial encoded proteins that comprise the individual oxidative phosphorylation complexes [3]. We have further shown that SAM treatment prevented these losses, particularly, select subunits of Complexes I and IV, which may explain, in part, how SAM maintains mitochondrial function and protects against the development of alcoholic liver injury [3, 18].

In this current study, we show for the first time that dietary supplementation with the methionine cycle metabolite, betaine, also protects against an ethanol-induced loss in oxidative phosphorylation system proteins. While the exact mechanism for this protection at the organelle level is not known, we propose that by preventing NOS2 induction and NO generation, betaine preserves the functioning of the electron transport chain, maintains the integrity of the liver, and protects against the development of alcoholic liver injury. Moreover, we propose that changes are associated with the normalization of hepatic SAM : SAH ratio and maintenance of methylation potential in response to betaine supplementation during chronic ethanol ingestion.

It is notable that NOS2 is absent in healthy, normal liver until it is transcriptionally activated by proinflammatory stimuli to produce large amounts of nitric oxide [27, 28]. Arguably, the chronic ethanol-mediated decrease in the SAM : SAH ratio in liver could be responsible for the ethanol-dependent increase in NOS2 expression, since studies have shown that impaired methylation increases NOS2 gene expression and nitric oxide [29]. Thus, lower hepatocellular SAM : SAH ratios would favor hypomethylation of the NOS2 promoter leading to increased NOS2 gene transcription and subsequent NOS2 protein increase and NO generation as seen in the current study (Figure 4). Conversely, it has also been shown that hypermethylation of the NOS2 gene promoter silences and downregulates NOS2 expression in foam cells [30]. In agreement with these results, we observed that concurrent supplementation with betaine in the ethanol diet suppressed ethanol-induced NOS2 gene and protein expression and NO generation. This finding suggests that betaine through maintenance of the SAM : SAH ratio and the methylation potential in liver could prevent the upregulation of NOS2 at the level of transcription.

Studies by Wu and Cederbaum have proposed that CYP2E1-mediated oxidative stress causes oxidative injury to the mitochondrion [31]. It has further been suggested that the alcohol-dependent increase in CYP2E1 may be partially related to decreased proteolysis of this protein in response to impaired proteasome function in the alcoholic liver [32]. Recently, we reported that the proteasome activity is directly impaired at ratios of SAM : SAH that correspond to those observed in livers of ethanol-fed rats [33]. Indeed, these results corroborate previous observations of a negative correlation between alcohol-induced increases in liver mRNA and protein levels of CYP2E1 with SAM : SAH ratios [34]. It should also be noted that SAM interacts and inhibits the catalytic activity of CYP2E1 in a reversible and noncompetitive manner *in vitro* [35]. However, despite elevated SAM levels and normal SAM : SAH ratios, CYP2E1 activity was only modestly inhibited (~12%) in rats fed the betaine-supplemented ethanol diets (Figures 5(a) and 5(b)). Regardless of elevated CYP2E1 in the livers of betaine-supplemented ethanol-fed rat, indices of steatosis nor defects in the mitochondrial respiratory chain was observed in these animals [14, 17, 19–23]. Taken together, these results suggest that the increased CYP2E1 activity is not sufficient to cause and the onset of liver steatosis or mitochondrial proteome defects.

Early studies demonstrated that chronic ethanol feeding specifically caused marginal decrease in total and a marked decrease in mitochondrial GSH levels compared to controls, which was associated with mitochondrial lipid peroxidation and progression of liver damage [36, 37]. Subsequently, studies showed that feeding SAM attenuated both ethanol-induced depletion of mitochondrial GSH and mitochondrial dysfunction [38]. Contrary to these studies, an alcohol-mediated increase in mitochondrial GSH has also been reported [3, 39]. Betaine treatment has been shown to restore alcohol-induced hepatic depletion of total GSH levels [40–43]. None of these studies, however, examined mitochondrial GSH status in particular following betaine treatment. In our study, we observe a significant decrease in total liver GSH (Figure 6(a)) but an (albeit small) increase in mitochondrial GSH in response to alcohol (Figure 6(b)) corroborating previous reports [3, 39]. We further observed that betaine treatment was unable to correct alcohol-induced changes in liver or mitochondrial GSH levels (Figures 6(a) and 6(b)). Our results suggest that neither the ethanol-induced defects nor the protective role of betaine on the various parameters examined in this study appears to be mechanistically related to the status of total or mitochondrial GSH level *per se*.

The present data reiterates that the normal mitochondrial proteome and function relies on the maintenance of methylation reactions. Indeed, supplementation with methyl donors, SAM [3, 4], and betaine (present study) at concentrations that maintain methylation reactions preserves mitochondrial proteome [3, 4] as well as prevents ethanol-dependent defects in mitochondrial respiration, mitochondrial ribosome dissociation, increases in mitochondrial superoxide production, and mitochondrial DNA damage [3, 4]. While the protective action of SAM and betaine at the level of the mitochondrion is now recognized, the mechanisms responsible for this protection are not clear. Similarly, how an alteration in the SAM : SAH directly or indirectly influences the composition of the mitochondrial proteome is undefined. Recent studies by Bailey and colleagues, using a comprehensive proteomics approach, demonstrate multiple ethanol and SAM specific alterations in key proteins involved in the oxidative phosphorylation system, as well as methionine, choline, and sulfur metabolism and chaperone systems [18]. Particularly, the SAM-dependent impacts on methionine and choline metabolism enzymes may be especially important, as these effects are predicted to protect methyltransferase reactions through boosting SAM levels. Similarly, the analyses on the oxidative phosphorylation system provided further support for an association among SAM, methylation, and respiratory chain maintenance as multiple subunits of the respiratory complexes were preserved in the SAM-supplemented ethanol group [18]. These findings are further validated in the current study, as we observed the ability of betaine to attenuate the alcohol-dependent loss in complex I and IV subunits. Methylation reactions have been reported to play an important role in the biosynthesis of lipoic acid, ubiquinone and biotin [44]; key cofactors of multiple cellular and mitochondrial enzyme systems. Taken together, future experiments will be directed at determining whether alcohol-dependent disruption in the activity of specific mitochondrial SAM-dependent methyltransferase(s) as a consequence of low SAM : SAH ratio is directly responsible for alterations in mitochondrion proteome and function.

In conclusion, this study shows for the first time that betaine prevents or blunts chronic ethanol-mediated alterations to the oxidative phosphorylation system proteome. We propose that the mitochondrial protection afforded by betaine is associated with the maintenance of hepatic SAM : SAH ratios and by blocking the ethanol-induced induction of NOS2 and NO, a key source of protein modification and damage. Moreover, this study demonstrates that proteomic-based methods like BN-PAGE are powerful tools to aid in the identification in the molecular targets of disease. Thus, maintenance of mitochondria function may be another key molecular target underlying the hepatoprotective effect of betaine against ethanol hepatotoxicity and fatty liver disease.

## Abbreviations

BN-PAGE: Blue native gel electrophoresis
CYP2E1: Cytochrome P450 2E1
GSH: Glutathione
NOS2: Inducible nitric oxide synthase
SAM: S-adenosylmethionine
SAH: S-adenosylhomocysteine.
